# Lipid and Amino Acid Pathway Metabolites Contribute to Cold Tolerance in *Quercus wutaishanica*

**DOI:** 10.3390/metabo13101094

**Published:** 2023-10-19

**Authors:** Qun Li, Yang Xu, Yan-Qun Liu, Li Qin

**Affiliations:** Department of Sericulture, College of Bioscience and Biotechnology, Shenyang Agricultural University, Shenyang 110866, China; liqun@syau.edu.cn (Q.L.); xuyang720r@163.com (Y.X.)

**Keywords:** amino acid, LC-MS/MS, low-temperature stress, phosphatidylcholine, *Quecus wutaishanica*

## Abstract

Cold is an important environmental stress affecting the growth, productivity, and geographic distribution of tree species. Oaks are important for environmental conservation and wood supplies. Oak metabolites respond to low temperatures (LTs). In this study, the physiological and metabolic responses of two oak species to cold stress were investigated and compared. The field observations and physiological responses showed that *Quercus wutaishanica* was more cold-tolerant than *Q. acutissima*. After frost, the one-year-old twigs of *Q. wutaishanica* had higher survival rates, accumulated more soluble sugar and protein, and exhibited higher superoxide dismutase (SOD) activity than those of *Q. acutissima*. Untargeted metabolomics identified 102 and 78 differentially accumulated metabolites in *Q. acutissima* and *Q. wutaishanica*, respectively, when the leaves were subjected to LTs (4 °C for 24 h). The carbohydrate and flavonoid metabolites contributed to the cold tolerance of both oak species. Succinate, an intermediate in the citric acid cycle, was significantly inhibited by LTs, a potential energy conservation strategy. Unlike *Q. acutissima*, *Q. wutaishanica* underwent metabolic reprogramming that significantly increased the contents of phosphatidylcholine, gallic acid, oxidized glutathione, shikimate, and phenylpyruvate under LTs. Our data provide a reference for characterizing the mechanisms involved in the response of oak species to cold temperatures and enhancing the cold tolerance of forest trees.

## 1. Introduction

The genus *Quercus* L. (oak), belonging to the family Fagaceae, is the most important woody genus in the Northern Hemisphere [1,2,3]. Oaks provide food, wood products, clothing, fuel, and building supplies [4]. Oak trees possess higher cold tolerance and can survive normally at −55 to −32 °C [5]. Fresh oak leaves are also used for silk production, as feed for Chinese oak silkworms (*Antheraea pernyi*) and Japanese oak silkworms (*A. yamamai*) [5]. In early spring, frost damage to oak leaves often causes significant economic losses to oak silkworm production in China [6]. An understanding of how oaks adapt to harsh environmental conditions is necessary to sustain productivity and meet future demands for commercial products. Through untargeted metabolomics analyses, 63–342 compounds were identified in *Quercus* under drought and cold stress, improving our understanding of the metabolic responses of oak trees to abiotic conditions in the current climate-change scenario [7,8,9]. Previous studies hypothesized that the deterioration of *Q. robur* was caused by freezing stress experienced during six months of storage at −7 °C, a decline in antioxidative potential, and the unsuccessful rerouting of the energy-production pathways [8]. However, knowledge regarding the cold tolerance mechanism of oak trees remains limited.

Low temperatures (LTs) are the primary abiotic factor limiting germination and affecting the growth habits, productivity, and geographical distribution of plants [10,11]. Cold acclimation refers to the process by which the plants acquire increased cold tolerance after being exposed to non-lethal LTs [12,13]. Several physiological and biochemical processes occur during cold acclimation, and many substances or protective proteins are synthesized, such as soluble sugars, proline, and cold-resistance proteins [14,15]. The exposure of plants to LTs can cause a decrease in the cell membrane fluidity, a rearrangement of the cytoskeleton, and an overproduction of reactive oxygen species (ROS), resulting in changes in the secondary metabolism [16]. At the metabolic level, LTs can cause an increase in phenolic compounds, including flavonoids, phenylpropanoids, anthocyanins, and lignins [17]. Free amino acids, which regulate nitrogen metabolism and mitigate chilling-induced oxidative damage, are also crucial in the response to LTs [18]. Cold acclimation has been extensively studied in rice, maize, and barley because LTs can significantly reduce plant yields [19]. An accurate metabolomic analysis of forest tree species is essential to understanding the fundamentals of physiological and biochemical stress and determining the potential mechanisms of cold tolerance [20]. However, research on the forest trees’ (including oaks) response to LTs has advanced more slowly compared to the analysis of model and crop species due to the trees’ long life cycles, large genome sizes, and lack of genomic tools [21].

*Quercus wutaishanica* and *Q. acutissima* are the two main species used in China to raise *A. pernyi* [5]. *Q. wutaishanica* is widely distributed in the northern part of the Qinling, Liupan, and Lüliang Mountains in China (31°41′ to 44°21′ N and 103°54′ to 129°32′ E) [22]. *Q. acutissima* is distributed in Hengduan Mountains, Yunnan-Guizhou Plateau, and Qin-Ba Mountains (18° to 42° N and 91° to 123° E) [23]. Field observations conducted for approximately 30 years at the Oak Silkworm Experimental Station of Shenyang Agricultural University (SYAU) have demonstrated that the two species have different levels of cold tolerance, with *Q. wutaishanica* being more cold-tolerant than *Q. acutissima*. This study focuses on the physiological and metabolomic responses to LTs in *Q. wutaishanica* and *Q. acutissima* grown in the same location. The metabolites of the two oak species in response to LTs stress are identified using untargeted metabolomics. The aim of this study is to attempt to explain the reason that *Q. wutaishanica* is more cold-tolerant than *Q. accutissima* by identifying differentially accumulated metabolites and analyzing metabolic pathways involved in *Q. wutaishanica*-specific cold responses. An in-depth understanding of cold responses can help safeguard forest productivity and contribute to understanding the cold resistance mechanism of oak trees.

## 2. Materials and Methods

### 2.1. Plant Materials

**Figure 1:**
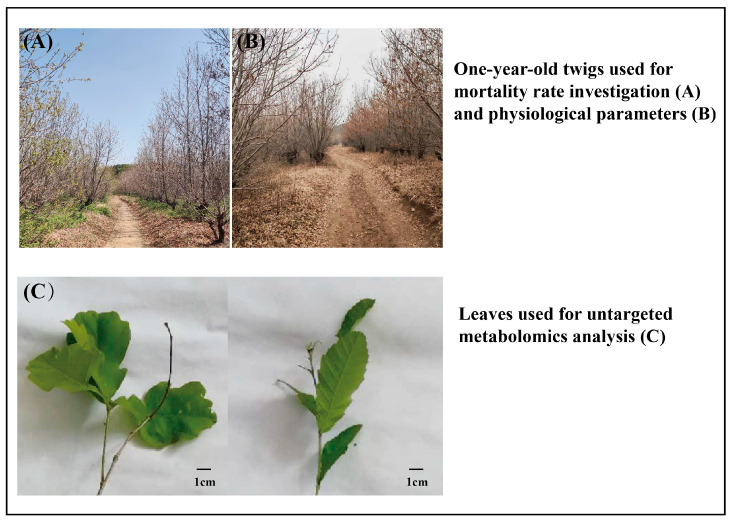
Samples used in this study. (**A**) *Q. wutaishanica* (**Left**) and *Q. acutissima* (**Right**) (Photo was taken on 4 April 2021); (**B**) *Q. wutaishanica* (**Left**) and *Q. acutissima* (**Right**) (Photo was taken on 1 December 2020); (**C**) Three-year-old seedling of *Q. wutaishanica* (**Right**) and *Q. acutissima* (**Left**).

The two oak species used in this study have been growing for about 60 years in the Oak Silkworm Experimental Field Station of SYAU, China (41°83′ N, 123°58′ E). To investigate the overwintering mortality of one-year-old twigs, we randomly sampled 30 individuals from each species on 4 April 2021 (Figure 1A). Three branches from each individual were chosen. We counted the number of all one-year-old twigs on each branch and recorded the number of dead twigs. The mortality rate was calculated as follows:

Mortality of one-year-old twigs (%) = (Number of dead twigs)/(Total number of twigs) × 100%

### 2.2. Analysis of Physiological Parameters

We collected 20 cm long, one-year-old twigs from 3 adult trees of each species on 1 December 2020, and the temperature was −12 to −1 °C (Figure 1B). The shoot segments were washed twice with distilled water, dried with filter paper, and sealed with paraffin wax at both ends. These samples were used to assess the physiological parameters.

The contents of soluble protein (SP), soluble sugar (SS), and malondialdehyde (MDA) were determined spectrophotometrically (UV2000, UNICO, Shanghai, China) as previously described [24]. Briefly, 0.5 g (fresh weight, FW) of the samples were ground and combined with 50 mmol/L of phosphoric acid buffer. After centrifugation at 12,000× *g* for 30 min at 4 °C, the supernatant was used to determine the SP content by measuring the absorbance at 595 nm.

The samples (0.5 g FW) were ground and combined with 15 mL of distilled water and boiled for 20 min. The supernatant was then used to determine the SS content by measuring the absorbance at 620 nm.

The samples (0.5 g FW) were ground and placed in an ice bath. A total of 1 mL of 0.25% thiobarbituric acid (TBA) was dissolved in 10% trichloroacetic acid, and the mixture was used to extract the MDA. The mixture was heated at 85 °C for 30 min and then rapidly chilled using ice. The pellets were removed after centrifugation at 4000× *g* for 10 min, and the specific absorbance values at 532 nm (the peak of the MDA-TBA complex) and 600 nm (nonspecific absorption) were determined. Finally, the MDA concentration was calculated using the extinction coefficient ε 532 155 mM^−1^ cm^−1^.

The activities of peroxidase (POD) and superoxide dismutase (SOD) were detected using the POD and SOD Kits (Jiancheng Bioengineering Institute, Nanjing, China), respectively. The samples (0.5 g FW) were ground, combined with ice-cold 0.1 mol/L phosphate buffer (pH 7.0), and centrifuged at 3500× *g* for 10 min. The supernatant was used to determine the POD and SOD activities.

### 2.3. Untargeted Metabolomics Analysis

Three-year-old seedlings of *Q. wutaishanica* and *Q. acutissima* planted in the same experimental field were used for metabolomics analysis (Figure 1C). Individuals of *Q. wutaishanica* and *Q. acutissima* with comparable height (10–15 cm) were transplanted into plastic pots (diameter × height: 20 × 13 cm) using the original soil, with one seedling per pot. The experiments were performed in July with initial average acclimatization temperatures of 30 and 20 °C (day/night) and relative humidity of 55% for two weeks. The individuals of the two species were randomly divided into two groups: one group was transferred to artificial chambers with temperature conditions of 25 °C (normal temperature (NT)), a light intensity of 800 µm m^−2^s^−1^, a photoperiod cycle of 12 h light and 12 h dark, and relative humidity of 75%; another group was placed in a refrigerator under conditions of alternating 12 h of artificial light and darkness (LT, 4 °C for 24 h). Six individuals were randomly selected from the two species, representing the NT and LT treatments. The first fully-unfolded leaves were collected immediately and washed with distilled water. These leaves were immediately frozen in liquid nitrogen and stored at −80 °C for the subsequent liquid chromatography-tandem mass spectrometry (LC-MS/MS) analysis. Six replications were performed for a total of 24 samples.

The leaves (100 mg FW) were ground and combined with liquid nitrogen, and 1000 μL of pre-cooled methanol-acetonitrile (ACN)-water (v:v:v = 2:2:1) was added. The mixture was subjected to an ultrasonic treatment in an ice bath for 60 min and then incubated at −20 °C for 60 min. After centrifugation at 16,000× *g* at 4 °C for 20 min, the supernatant was removed and vacuum-dried at −80 °C. The dried extract was dissolved in 100 μL of 50% ACN solution and centrifuged at 14,000× *g* at 4 °C for 15 min. The supernatant was removed for the subsequent LC-MS/MS (Bioprofile Co. Ltd., Shanghai, China).

Metabolomics profiling was conducted using ultra-performance liquid chromatography-electrospray ionization-quadrupole time-of-flight mass spectrometry (UPLC-ESI-Q-TOF-MS) (UHPLC, 1290 Infinity LC, Agilent Technologies, Santa Clara, CA, USA) coupled with TripleTOF 5600 (AB Sciex, Framingham, MA, USA). For the hydrophilic interaction liquid chromatographic (HILIC) separation, the samples were analyzed using a 2.1 mm × 100 mm ACQUIY UPLC BEH 1.7 μm column (Waters, Wexford, Ireland). The flow rate was 0.3 mL/min, and the mobile phase contained 25 mM of ammonium acetate and 25 mM ammonium hydroxide in water (A) and ACN (B). The gradient was 95% B for 0.5 min; it was linearly reduced to 65% in 6.5 min and to 40% in 2 min. It was then maintained for 1 min and increased to 95% in 1.1 min, with a 5 min re-equilibration period. Electrospray ionization (ESI) in positive and negative modes was used for MS data acquisition. The ESI source conditions were as follows: ion source gas 1, 60; ion source gas 2, 60; curtain gas, 30; source temperature, 600 °C; and ion spray voltage floating (ISVF) ± 5500 V. In the MS-only acquisition, the instrument acquired data in the *m*/*z* range of 60–1200 Da, and the time for TOF MS scanning was 0.15 s/spectra. In the auto MS/MS acquisition, the instrument acquired data in the *m*/*z* range of 25–1200 Da, and the time for the product ion scan was 0.03 s/spectra. The product ion scan was conducted using information-dependent acquisition with high sensitivity. The collisional energy was fixed at 30 V with ±15 eV. The declustering potential was ± 60 V. Quality control (QC) samples were prepared by pooling aliquots of all samples representative of the analyzed samples and used for data normalization. Blank samples (75% ACN in water) and the QC samples were injected every six samples during the acquisition.

The raw MS data were converted to MzXML files using ProteoWizard MSConvert [25] and processed using XCMS [26] for feature detection, retention-time correction, and peak area extraction. The metabolomic data were analyzed using MetaboAnalyst 4.0 (https://www.metaboanalyst.ca/, accessed on 20 March 2023) [27]. The metabolite classification was performed using the Human Metabolome Database (HMDB) (http://www.hmdb.ca, accessed on 20 March 2023) [28,29,30]. The key expressed metabolites were filtered by the variable importance in projection (VIP) scores using rules used in previous research [31].

The KEGG pathway database (https://www.kegg.jp/kegg/pathway.html, accessed on 20 March 2023) has records of metabolic reactions and concatenates possible metabolic pathways and regulatory proteins [32]. KEGG pathway analysis of the differential metabolite data was used to identify the perturbed biological pathways. KEGG enrichment analyses were carried out with Fisher’s exact test, and false discovery rate (FDR) correction of multiple tests was performed.

### 2.4. Statistical Analysis

All statistical analyses were performed using SPSS 25.0 software (SPSS Inc., Chicago, IL, USA). The differences between the groups were compared using independent *t*-tests. *p*-values < 0.05 indicated statistical significance.

## 3. Results

### 3.1. Overwintering Mortality Ratio and Physiological Responses of Q. acutissima and Q. wutaishanica to LTs

**Figure 2:**
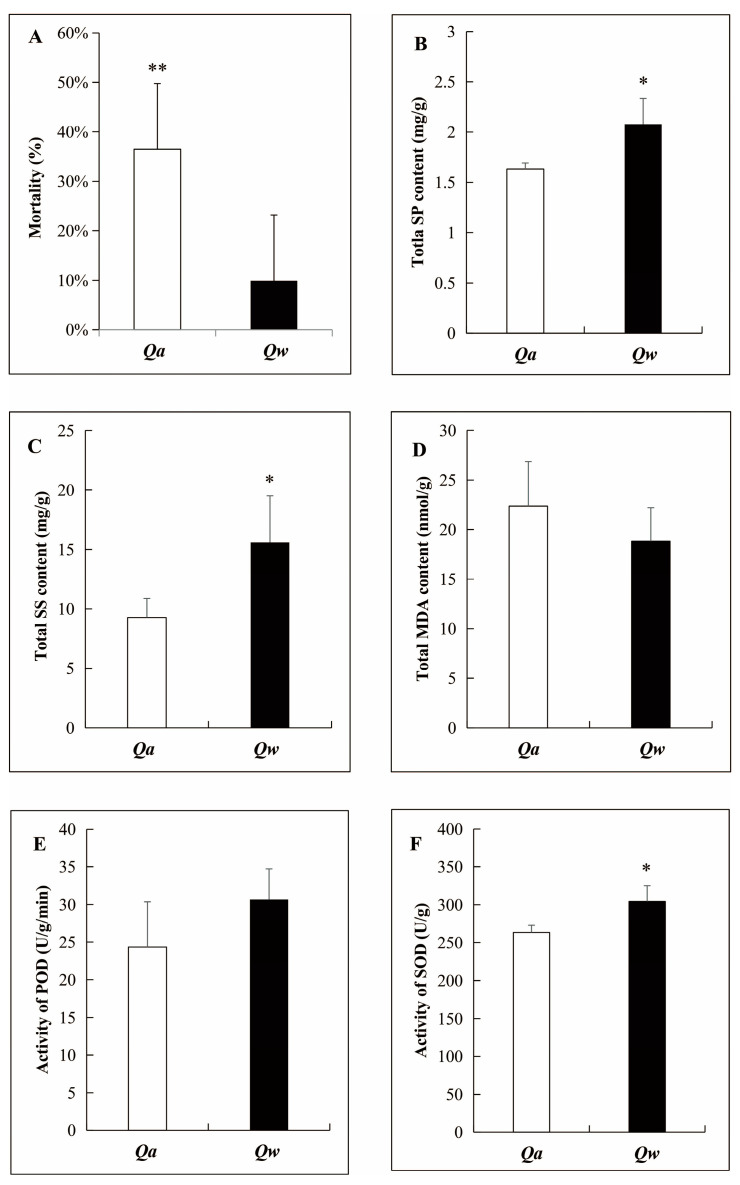
Comparison of overwintering mortality and physiological parameters of one-year-old shoots of *Q. acutissima* and *Q. wutaishanica*. (**A**) Overwintering mortality; (**B**) Soluble protein (SP) content; (**C**) Soluble sugar (SS) content; (**D**) Malondialdehyde (MDA) content; (**E**) Peroxidase (POD) activity; (**F**) Superoxide dismutase (SOD) activity. Error bars indicate ± SD (A, *n* = 90; B–F, *n* = 3). *, *p* < 0.05; **, *p* < 0.01.

The overwintering survival rate of one-year-old twigs in two oak species was more than 60%. However, the mortality ratio was significantly higher for *Q. acutissima* (36.46 ± 13.29%) than for *Q. wutaishanica* (9.91 ± 6.27%) (Figure 2A), confirming the field observation that *Q. wutaishanica* was more cold-tolerant than *Q. acutissima*.

Some physiological parameters were measured using one-year-old twigs as samples. No significant difference in the MDA content (Figure 2D) and the POD activity (Figure 2E) of the one-year-old twigs was observed between *Q. acutissima* and *Q. wutaishanica* exposed to −7 °C. However, the SP and SS contents and the SOD activity were significantly higher in *Q. wutaishanica* than in *Q. acutissima* (Figure 2B,C,F). This result confirmed that *Q. wutaishanica* was more cold-tolerant than *Q. acutissima*.

### 3.2. Detection of Metabolites by LC-MS/MS

**Figure 3:**
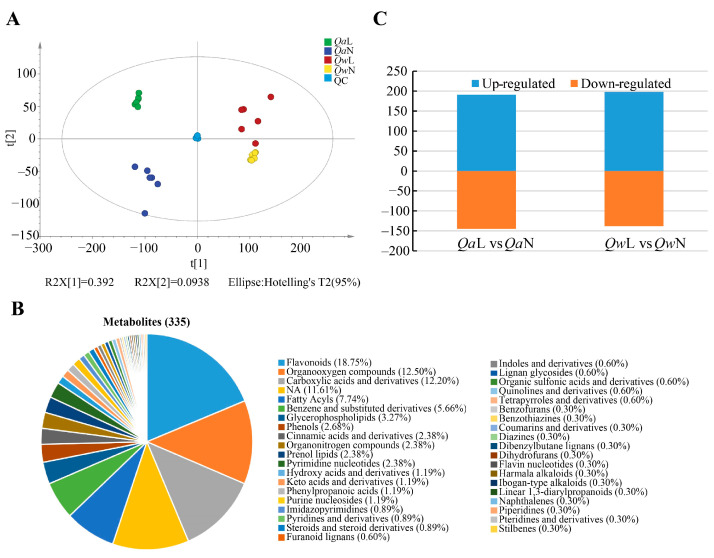
Metabolite data obtained from LC-MS/MS. (**A**) Results of principal component analysis of *Quercus* leaf metabolites under LTs (4 °C) and NTs (25 °C); (**B**) Metabolite classification of *Quercus*; (**C**) Metabolites of *Quercus* in response to LTs (4 °C) and NTs (25 °C).

The overlap of the QC sample detection curves was high and the QC samples were clustered closely, indicating good repeatability and reliability of the MS data. The PCA model was obtained through 7-fold cross-validation (Figure 3A). A total of 335 metabolites were detected in *Q. acutissima* and *Q. wutaishanica*, including 40 different substances, such as organooxygen compounds, flavonoids, carboxylic acids and derivatives, and benzene and substituted derivatives (Table S1). The flavonoids (18.75%), organooxygen compounds (12.50%), carboxylic acids and derivatives (12.20%), and fatty acyls (7.74%) were the most abundant in the two oak species (Figure 3B). The dynamic up- and down-regulation of the detected metabolites of the two oak species exposed to the LT and NT treatments were analyzed. A total of 191 and 197 metabolites were up-regulated in the LT treatment in *Q. acutissima* and *Q. wutaishanica*, respectively (Figure 3C).

**Figure 4:**
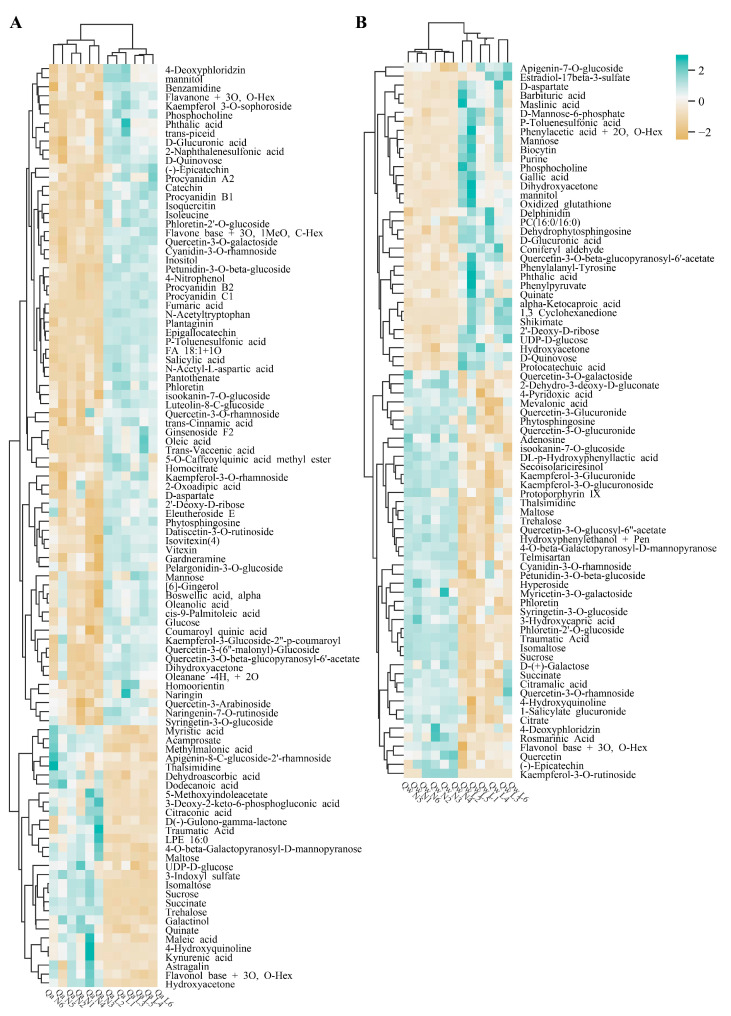
Results of hierarchical clustering analysis (HCA) of differentially accumulated metabolites in *Q. acutissima* and *Q. wutaishanica* under LTs. (**A**) *Q. acutissima*; (**B**) *Q. wutaishanica*. The columns represent the six replicates for the LT and NT treatments. The colors correspond to log2-transformed fold changes. An increase or decrease in the metabolite level is indicated by increases in the intensities of blue and yellow, respectively. 1–6: One to six individuals, respectively.

The relative metabolite levels of *Q. acutissima* and *Q. wutaishanica* in the two treatments were compared. Differentially accumulated metabolites (DAMs) were identified based on the VIP score (>1.0) and the Mann–Whitney *p*-value (<0.05) to determine differences in the metabolic profiles in response to LT between the two oak species. A total of 102 and 78 differentially expressed metabolites were accumulated under LTs in *Q. acutissima* and *Q. wutaishanica*, respectively (Figure 4, Tables S2 and S3). In *Q. acutissima*, the most highly increased metabolites were N-acetyltryptophan (18.1×), Fumaric acid (17.7×), Flavone base + 3O, 1MeO, C-Hex (5.0×), Isoleucine (5.0×), N-acetyl-L-aspartic acid (4.7×), and Plantaginin (4.6×) (Figure 4A, Table S2). In *Q. wutaishanica*, the abundance of some metabolites increased substantially, including Mannose (6.4×), Biocytin (5.3×), Purine (5.0×), Maslinic acid (4.6×), Phthalic acid (4.5×), and Dihydroxyacetone (4.5×) (Figure 4B, Table S3).

**Figure 5:**
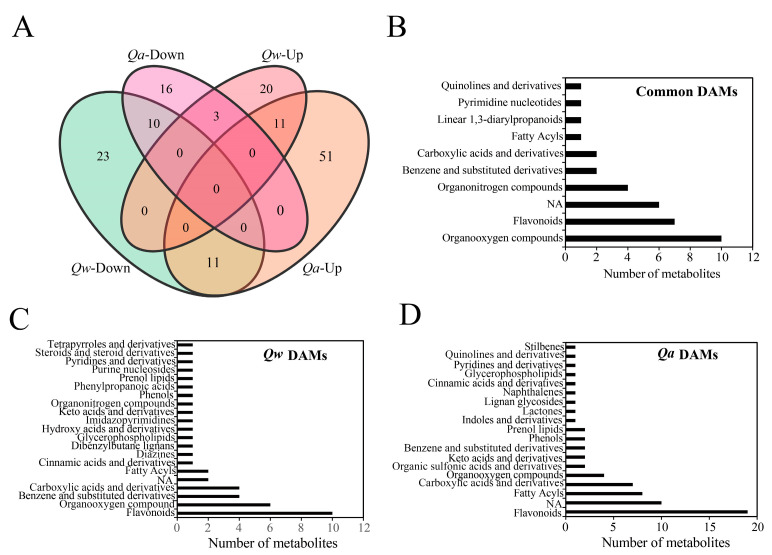
Metabolomic responses to LT treatment. (**A**) Venn diagrams of differentially accumulated metabolites; (**B**) Common differentially accumulated metabolites; (**C**) Specific differentially accumulated metabolites of *Q. wutaishanica*; (**D**) Specific differentially accumulated metabolites of *Q. acutissima*.

We then compared the up- and down-regulated sets of metabolites to obtain insights into the shared and divergent mechanisms in *Q. acutissima* and *Q. wutaishanica*. A total of 35 DAMs were common to the two oak species under LTs, including 11 up-regulated metabolites (e.g., 2′-Deoxy-D-ribose, D-aspartate, D-Glucuronic acid, Dihydroxyacetone, D-Qunovose, Mannitol, Mannose, Phosphocholine, Phthalic acid, P-Toluenesulfonic acid, and Quercetin-3-O-beta-glucopyranosyl-6’-acetate) and 10 down-regulated metabolites (4-Hydroxyquinoline, 4-O-beta-Galactopyranosyl-D-mannopyranose, Flavonol base + 3O, O-Hex, Isomaltose, Maltose, Succinate, Sucrose, Thalsimidine, Traumatic acid, and Trehalose). These 35 DAMs might be associated with the same metabolic regulatory pathways for cold response in oaks (Figure 5A, Tables S2 and S3). A total of 34 DAMs were up-regulated in *Q. wutaishanica*, among which 14 metabolites overlapped between *Q. acutissima* and *Q. wutaishanica* (Figure 5A, Tables S2 and S3). Interestingly, three metabolites were up-regulated in *Q. wutaishanica* but down-regulated in *Q. acutissima* (Hydroxyacetone, Quinate, and UDP-D-glucose). Additionally, 67 DAMs were specific to *Q. acutissima*, whereas 43 DAMs were specific to *Q. wutaishanica*.

A total of seven flavonoids were detected among the thirty-five DAMs common to both oak species (Figure 5B). Also, flavonoids were the most abundant DAMs, with 10 and 19 flavonoids observed in *Q. wutaishanica* and *Q. acutissima*, respectively (Figure 5C,D).

### 3.3. Pathway Enrichment and Topological Analysis of Differentially Accumulated Metabolites in the Two Oak Species

**Figure 6:**
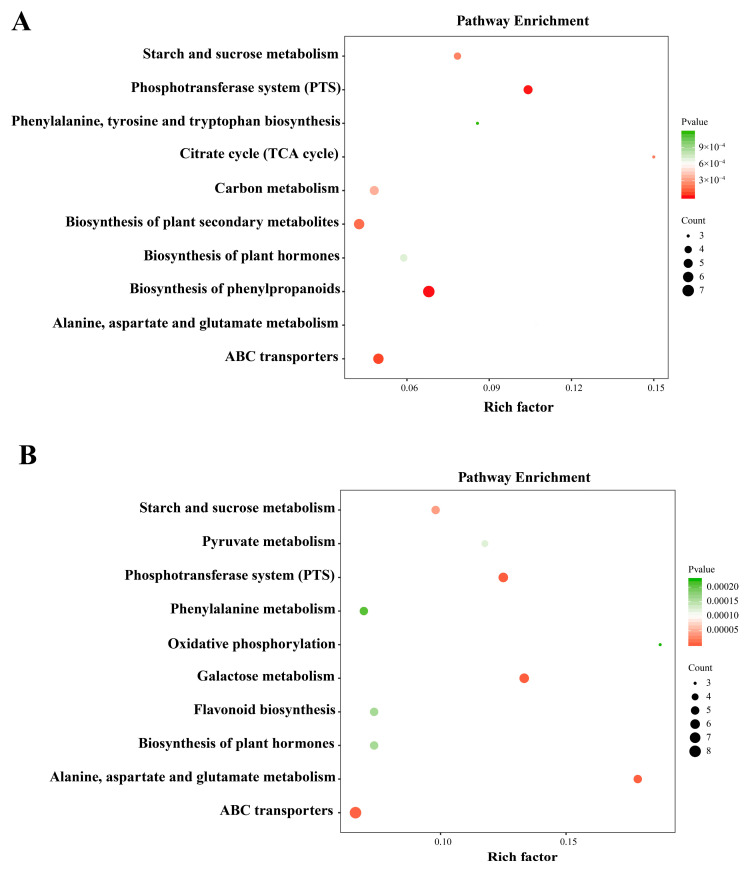
Results of KEGG pathway analysis of differentially accumulated metabolites. (**A**) Enriched KEGG pathways for *Q. wutaishanica*; (**B**) Enriched KEGG pathways for *Q. acutissima*. The matched pathways are indicated by the circles. The Rich factor is the ratio of the number of metabolites in the corresponding pathway to the total number of metabolites detected and annotated in the pathways. The color of the circles indicates the *p* value (darker colors indicate more significant changes in the metabolites in the pathway). The circle size corresponds to the pathway impact scores.

The KEGG pathway enrichment analysis was performed to characterize the metabolic pathways involved in the two oak species’ responses to cold temperatures. In *Q. wutaishanica*, the DAMs were significantly enriched (*p* < 0.05) in eight pathways associated with lipid metabolism (glycerolipid and alpha-linolenic acid metabolisms), carbohydrate metabolism (starch and sucrose metabolism, pentose and glucuronate interconversions, galactose metabolism, and amino sugar and nucleotide sugar metabolism), and amino acid metabolism (phenylalanine, tyrosine, and tryptophan biosynthesis) (Figure 6A). In *Q. acutissima*, the DAMs were assigned to six pathways related to lipid metabolism (fatty acid biosynthesis and ascorbate and aldarate metabolisms), carbohydrate metabolism (propanoate and galactose metabolisms), and amino acid metabolism (tryptophan metabolism) (Figure 6B).

**Figure 7:**
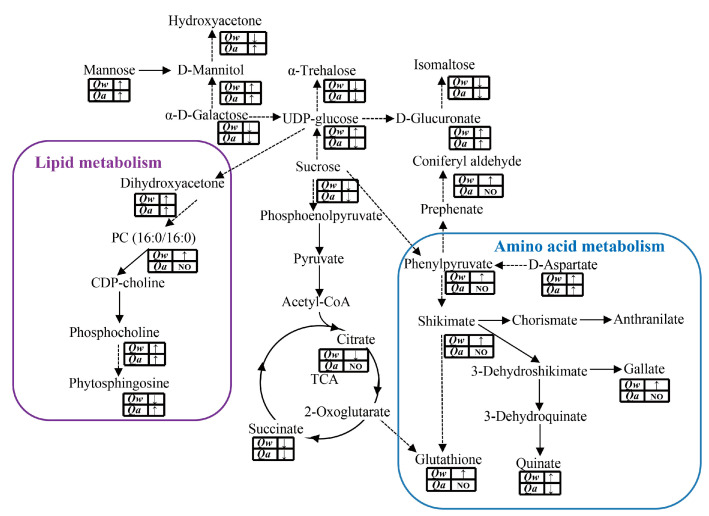
Changes in the metabolites involved in carbohydrate, amino acid, and lipid metabolism under LT stress. The purple rounded rectangle represents the lipid metabolism, and the blue rounded rectangle represents the amino acid metabolism. The solid and dashed arrows indicate single- and multi-step reactions, respectively. The “↑” and “↓” represent up- and down-regulated, respectively.

As a central biochemical pathway, the tricarboxylic acid (TCA) cycle was significantly inhibited by LT stress. Specifically, the rates of two intermediates in the TCA cycle, citrate and succinate, decreased significantly in *Q. wutaishanica*. However, only the rate of citrate decreased significantly in *Q. acutissima* (Figure 7). In contrast, there was a significant accumulation of multiple metabolites (α-trehalose, UDP-D-glucose, D-glucuronate, Sucrose, mannitol, Hydroxyacetone, Isomaltose, Mannose, and α-D-galactose) involved in the carbohydrate metabolism in both oak species (Figure 7).

Dihydroxyacetone, phytosphingosine, and phosphocholine, involved in the lipid pathway, were significantly enriched in both oak species in response to LTs. However, phosphatidylcholine (PC) was significantly enriched only in *Q. wutaishanica*, indicating higher cold tolerance of this species (Figure 7).

Some secondary metabolites (quinate, shikimate, phenylpyruvate, gallate, glutathione, and D-aspartate) derived from amino acid metabolism were significantly enriched in *Q. wutaishanica* in response to LTs. However, shikimate, gallate, glutathione, and phenylpyruvate were unaffected in *Q. acutissima* (Figure 7).

## 4. Discussion

### 4.1. Q. wutaishanica has Higher Cold Tolerance Than Q. accutissima

*Q. wutaishanica* is found more than two degrees of latitude further north than *Q. acutissima* in China, suggesting that *Q. wutaishanica* may be more cold-tolerant than *Q. accutissima*. The field observations and the overwintering mortality of one-year-old twigs confirmed that *Q. wutaishanica* was more cold-tolerant than *Q. acutissima*.

Plants acclimate to cold stress by accumulating osmotic regulators, such as SS, proline (Pro), and SP [33]. Under cold stress, plants exhibit a decrease in MDA content, with an increase in the activities of antioxidant enzymes, such as SOD and POD, and eliminate excess ROS to alleviate the oxidative damage in plant cells [34]. The MDA content and POD activity in *Q. wutaishanica* and *Q. acutissima* were not significantly different in this study. However, the SS and SP contents and the SOD activity of *Q. wutaishanica* were significantly higher than that of *Q. acutissima* in the LT treatment. This result is consistent with that of Oberschelp et al. [20], who observed that *Eucalyptus benthamii*, a frost-tolerant species, showed the highest total SS content among three Eucalyptus species. Du et al. [35] also found that the SOD activity was higher in a chilling-tolerant rice variety (Qiutianxiaoting) than in a chilling-susceptible variety (93-11). Similar results were observed in oaks, as the SP and SS levels and SOD activity of four oak species (*Q. serrata*, *Q. fabri*, *Q. variabilis*, and *Q. acutissima*) all showed an increasing trend in the early stages of drought stress [24]. Overall, our results demonstrated that *Q. wutaishanica* was relatively more cold-tolerant compared to *Q. acutissima*.

### 4.2. Universal Cold Resistance Mechanisms of Oaks

In this study, the two oaks’ overwintering survival rates of one-year-old twigs were higher than 60%, confirming the higher cold tolerance of the two oaks. Plants make different defense mechanisms in response to cold stress. One common way is to produce metabolites involved in carbohydrate metabolism, the TCA cycle, amino acids metabolism, and flavonoid biosynthesis pathways [36]. In this study, some carbohydrates were significantly enriched in the two oak species under LT conditions, which is in line with previous studies showing that soluble carbohydrate concentrations were closely related to cold-hardiness in *Q. robur*, *Q. pubescens*, and *Q. ilex* [37]. Mannose can be obtained by the oxidation of mannitol [38]; mannitol is an osmolyte and solute that provides resistance against various abiotic stresses in higher plants [38]. Mannose was significantly enriched in the two oak species, similar to *Dendrobium catenatum* under cold stress [39].

These results showed that the biosynthesis of amino acids relieves stress in plants, as previously reported [31,40,41]. Although no proline was detected in *Q. acutissima* and *Q. wutaishanica*, aspartate was significantly enriched in both oak species. This result was in line with previous studies that found aspartate was enriched in *Haberlea rhodopensis*, *Thellungiella halophyla*, and *Arabidopsis thaliana* during cold acclimation [42]. Quinate, involved in phenylalanine, tyrosine, and tryptophan biosynthesis, was also significantly enriched in the two oak species.

The TCA cycle generates energy and maintains normal plant growth; it is an important primary metabolic pathway. In this study, the contents of two TCA cycle intermediates, citrate and succinate in *Q. wutaishanica* and succinate in *Q. acutissima*, were significantly lower under LT stress than in NT. This result was consistent with previous studies in which cold stress inhibited the TCA cycle in *Arabidopsis* [43,44] and *Euphorbia fischeriana* [45]. However, contrasting data for pepper showed that most genes and metabolites involved in the TCA cycle were up-regulated in cold-tolerant plants under cold stress [46]. The lower content of the TCA cycle intermediates in the two oak species observed in this study might indicate an energy conservation strategy to cope with LT stress. Particularly, the succinate content was significantly lower in the LT treatment in the two oak species. Succinate plays a crucial role in the generation of adenosine triphosphate (ATP) in mitochondria [47]. The lower content of succinate may indicate an increased demand for carbon sources and respiratory-energy-generation to activate growth. The regulatory relationship between the significantly lower succinate content and cold resistance in oaks requires further research.

Flavonoids are secondary metabolites acting as antioxidants in plants. They accumulate during stressful conditions. The total flavonoid content was affected by ozone and water stress in *Q. ilex*, *Q. pubescens*, and *Q. robur*. It can be considered a biochemical trait to improve the adaptability of plants to harsh environments [48]. Stress caused by temperature decreasing to 4–10 °C is conducive to the accumulation of flavonoids in plants [36]. Following these results, flavonoids were the most common DAMs in the two oak species at 4 °C. In summary, the up-regulation of sugars, amino acids, TCA cycle intermediates, and flavonoids might be responsible to explain the tolerance in the two species, as in other plants.

### 4.3. Lipid and Amino Acid Pathway Metabolites May Contribute to Cold Tolerance in Q. wutaishanica

Our study found that many metabolic changes were unique to *Q. wutaishanica,* which may explain why *Q. wutaishanica* was more cold-tolerant than *Q. wutaishanica*. The metabolites glutathione, shikimate, gallic acid, and phenylpyruvate, involved in the metabolic pathway of amino acids, were significantly higher in *Q. wutaishanica* under LT conditions. Glutathione participates in various physiological processes in plants [49]. Plants overexpressing CPK24 exhibited increased accumulation of glutathione during cold stress [49]. Chen et al. [50] also observed that chilling stress enhanced the oxidized glutathione content in six-day-old wheat seedlings subjected to 4 °C for 24 h. In *Q. wutaishanica*, the abundance of glutathione increased (1.5×) under LT but was not detected in *Q. acutissima.* The accumulation of glutathione in *Q. wutaishanica* may help increase the ROS-scavenging ability of *Q. wutaishanica* to balance the effect of cold damage.

Shikimate and gallic acid are intermediates in the phenylpropanoid biosynthesis pathway, and phenylpropanoid is a critical component in the amino acid pathway. Shikimate and gallic acid are associated with anthranilate biosynthesis. Anthranilic acid is a key metabolite that provides energy and metabolic materials for rice seed germination under LTs [31]. Aromatic amino acids in plants act as precursors for critical compounds, such as hormones, electron carriers, antioxidants, and enzyme cofactors [51]. A significant increase in these aromatic amino acids depended on an increase in the shikimate level at LTs (day/night: 18/10 °C for 7 d) in maize [52]. Previous studies have shown that gallic acid is an antioxidant acting as a neuroprotector in different models of neurodegeneration, neurotoxicity, and oxidative stress [53]. A marked elevation of the gallic acid content was also observed in lentil sprouts at 4 °C and 40 °C [54]. In this study, the shikimate (3.1×) and gallic acid (1.9×) accumulated specifically in the cold-tolerant *Q. wutaishanica*, indicating that phenylpropanoid biosynthesis may play a positive regulatory role in the cold tolerance of *Q. wutaishanica*.

Plants utilize phenylpyruvate pathway to produce phenylalanine (Phe) [55]. Phe is a precursor for numerous plant compounds that are crucial for plant defense against different types of stresses, including LTs [56]. Although Phe was detected in both *Q. wutaishanica* and *Q. acutissima*, there was no significant difference between LT and NT. Our results showed that the enrichment in phenylpyruvate (3.6×) observed in *Q. wutaishanica* may contribute to the higher cold tolerance of this species.

Phosphatidylcholine (PC) is an intermediate product in the lipid metabolism pathway. The major role of PC is stress adaptation in plants [57]. The pool of PC might be tightly regulated and is critical to maintain cell structure and function under stress. The expression level of PC-synthesis-pathway genes correlates with the modification of the PC biosynthesis rate and responds to temperature stress [57]. Our results showed that the PC content in *Q. wutaishanica* was 1.6-fold higher in the LT treatment than in the NT treatment; however, it was not detected in *Q. acutissima*. The high level of PC content could function to maintain cell structure and improve the ability of *Q. wutaishanica* to adapt to LTs.

Our work demonstrated that *Q. wutaishanica* was more cold-tolerant than *Q. acutissima*. Metabolites involved in the biosynthesis of amino acids and lipids contributed to the cold tolerance of *Q. wutaishanica*. However, the results of the untargeted metabolomics analysis were insufficient to explain the attributes of the oak species. Metabolites are the final products, and genes regulate their generation and accumulation. Therefore, a transcriptome analysis of oak species under cold stress should be conducted to obtain deeper insights into the gene-level processes.

## 5. Conclusions

In this study, we investigated the physiological and metabolic responses of *Q. wutaishanica* and *Q. acutissima* to LTs. Compared with the latter, *Q. wutaishanica* exhibited higher overwintering survival rates, SOD activity, and content of SS and SP in one-year-old twigs, establishing that its cold tolerance was higher than *Q. acutissima*. The metabolic profiles indicated that the metabolism of carbohydrates, amino acids, TCA cycle intermediates, and flavonoids contribute to the common cold responses of the two oak species. Moreover, the metabolites related to lipid and amino acid biosynthesis help mediate *Q. wutaishanica* cold tolerance. Our results provide useful information for understanding the mechanisms underlying the cold tolerance of oak trees.

## Data Availability

Mass spectrometry data with identifier No. MTBLS6958 have been uploaded to Metabolights (www.ebi.ac.uk/metabolights/MTBLS6958). All data generated or analyzed during this study are included in this published article.

## Supplementary Materials

The following supporting information can be downloaded at https://www.mdpi.com/article/10.3390/metabo13101094/s1: Table S1, Detected metabolites of *Q. acutissima* and *Q. wutaishanica* in the LT and NT treatments; Table S2, Differentially expressed metabolites of *Q. acutissima* in the LT and NT treatments; Table S3. Differentially expressed metabolites of *Q. wutaishanica* in the LT and NT treatments.

## Abbreviations

LT, low temperature; NT, normal temperature; QC, quality control; *Qw*, *Quecus wutaishanica*; *Qa*, *Q. acutissima*; *Qw*L, *Q. wutaishanica* under LT; *Qw*N, *Q. wutaishanica* under NT; *Qa*L, *Q. acutissima* under LT; *Qa*N, *Q. acutissima* under NT; TCA cycle, citric acid cycle; LC-MS/MS, liquid chromatography-tandem mass spectrometry; SP, soluble protein; SS, soluble sugar; MDA, malondialdehyde; POD, peroxidase; SOD, superoxide dismutase.
